# Late-Life Depressive Symptoms, Religiousness, and Mood in the Last Week of Life

**DOI:** 10.1155/2012/754031

**Published:** 2012-07-15

**Authors:** Arjan W. Braam, Marianne Klinkenberg, Henrike Galenkamp, Dorly J. H. Deeg

**Affiliations:** ^1^Longitudinal Aging Study Amsterdam, Department of Epidemiology and Biostatistics, EMGO Institute for Health and Care Research, VU University Medical Center Amsterdam, Van der Boechorststraat 7, 1081 BT Amsterdam, The Netherlands; ^2^Department of Emergency Psychiatry & Department of Specialist Training, Altrecht Geestelijke Gezondheidszorg, Utrecht, The Netherlands; ^3^Integraal Kankercentrum Nederland, Amsterdam, The Netherlands

## Abstract

Aim of the current study is to examine whether previous depressive symptoms modify possible effects of religiousness on mood in the last week of life. After-death interviews with proxy respondents of deceased sample members of the Longitudinal Aging Study Amsterdam provided information on depressed mood in the last week of life, as well as on the presence of a sense of peace with the approaching end of life. Other characteristics were derived from interviews with the sample members when still alive. Significant interactions were identified between measures of religiousness and previous depressive symptoms (CES-D scores) in their associations with mood in the last week of life. Among those with *previous* depressive symptoms, church-membership, church-attendance and salience of religion were associated with a greater likelihood of depressed mood in the last week of life. Among those *without* previous depressive symptoms, church-attendance and salience of religion were associated with a higher likelihood of a sense of peace. For older adults in the last phase of life, supportive effects of religiousness were more or less expected. Fore those with recent depressive symptoms, however, religiousness might involve a component of existential doubt.

## 1. Introduction

One important aspect of religion is how it may guide people through questions about the end of life. For some religious believers, it is clear that death only implies a transition. Others are less convinced, and may doubt about the existence of a transition, or about the conclusion of a judgement on their moral behaviour. In a previous study, we focused on the role of religiousness with respect to aspects of mood in the last week of life, as observed in a sample of older adults in The Netherlands [1]. Several aspects of religiousness were included, but none of them was associated with depressed mood in the last week of life, as reported by surviving relatives. Nonetheless, church attendance earlier in life predicted a “sense of peace” with the approaching end of life. Therefore, only modest support was found for the adaptive potential of religion in the last week of life. A possibly maladaptive aspect was not identified in this first report. Furthermore—although the analyses were adjusted for effects of previous depressive symptoms—the first study did not focus on those who were prone to depression during their lifetime.

The Netherlands represents a highly secularized country, but the older generation has still grown up in a society in which religious traditions had a prominent role, and many older people still endorse religious beliefs [2]. An ongoing debate in The Netherlands, especially among psychologists of religion and mental health professionals, is about the question whether religious beliefs, instead of giving support, may provoke depressive symptoms, such as feelings of guilt [3]. Indeed, for older people with a depressive syndrome, feelings of guilt were more often reported for the Calvinist Protestants and Roman Catholics, compared to nonchurch members [4]. The same was true for complaints of psychomotor inhibition, especially among depressed Protestants, but among the nondepressed, there were no denominational differences in guilt or psychomotor inhibition. Therefore, the relationship between facets of religiousness and mood seems to differ between the depressed and the nondepressed.

The last phase of life may follow different trajectories, such as with a gradual or rapid physical decline, and the mental demands will vary across the different types of illness. The last phase of life is characterized by inevitable adjustments for many older adults. There is a large need of informal and formal care, and many have to face a transfer to a different living environment [5]. In a recent study from the US, Hui and colleagues described fairly high levels of spiritual distress (such as feeling despair and brokenness, from an existential point of view) among patients with advanced cancer [6]. As expected, spiritual distress was associated with depression.

On the other hand, several studies among terminally ill patients showed associations between Spiritual Well-being Scale scores and lower levels of psychological distress [7, 8]. As some content overlap may occur between spiritual well-being and emotional well-being (or its reverse, psychological distress), these studies included statistical adjustment for depressive symptoms. Which aspects of religiousness and spirituality specifically determined the association with psychological distress in the terminally ill is difficult to say because the measure of spiritual well-being combines several aspects. One study included the belief in a hereafter as a distinct variable, and this was associated with lower levels of hopelessness, but not with feelings of anxiety or depression [9]. A complementary finding by Van Laarhoven and colleagues, in a small sample of advanced cancer patients, was the association of an explicit agnostic perspective on death and afterlife with higher levels of hopelessness [10]. The authors also described a negative association, but only at a nonsignificant level, between an explicit religious attitude and depression. In a palliative care study, a significant negative association with depression or anxiety disorder was found for church-attendance, but not for religious affiliation, prayer, or subjective religiousness [11]. 

With respect to the association between religiousness and the course of depression, several studies (from the US, Netherlands, and Australia), have shown that intrinsic religious motivation (or salience of religion) was associated with a quicker remission of the depression [12]. Findings in the literature about the association between church-attendance and the course of depression are however less consistent [13].

Clinical experience and epidemiological evidence have made clear that depression and depressive symptoms (or “subthreshold” depression) tend to recur, and for a minority, to persist, also in later life [14–16]. Therefore, the best predictor of depression is previous depression, and likewise, we expect that the vulnerability to depression will also predict depressed mood in terminal patients. With respect to the possible role of religiousness in this last phase of life, little is known about how an existing vulnerability to depression interferes with supportive or undermining effects of religiousness.

The current, population-based study focuses on relationships between aspects of religiousness and mood in the last week of life, as reported by surviving relatives of deceased sample members of the Longitudinal Aging Study Amsterdam (LASA) [17]. Information on religiousness was also obtained from the LASA respondents who were interviewed during lifetime about several aspects of religious life, as well as about depressive symptoms. In our previous study, we found that previous depressive symptoms predicted depressed mood, anxiety, and lack of sense of peace in the last week of life [1]. The current study aims to examine whether previous depressive symptoms modify associations between aspects of religiousness and mood in the last week of life, either giving way to a supportive potential of religiousness (e.g., for salience of religion), or to maladaptive effects (e.g., for certain convictions such as belief in hell).

## 2. Methods

### 2.1. Sample

**Figure 1:**
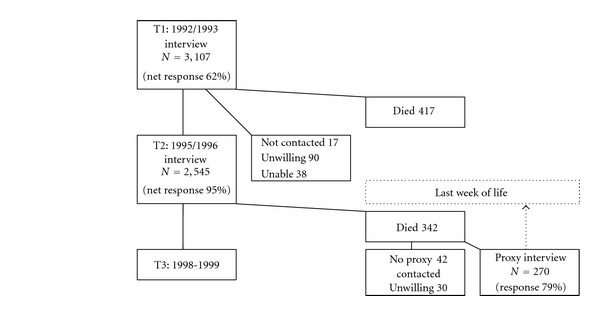
Flowchart of sampling times of the proxy interviews of deceased respondents of the Longitudinal Aging Study Amsterdam between T2-T3.

The Longitudinal Aging Study Amsterdam (LASA) is an ongoing interdisciplinary study on predictors and consequences of changes in autonomy and well-being in the aging population. The LASA cohort is based on a nationwide random sample of older adults between the ages of 55 and 85, stratified for age, sex, and expected mortality five years into the study. Registries of 11 municipalities in areas in the West (mostly secularized, including Amsterdam), North-east (predominantly Protestant), and South (predominantly Roman Catholic) of The Netherlands provided the sampling frame [18, 19]. The realized number of respondents in the LASA baseline interview cycle in 1992/1993 amounted to 3,107. Respondents were interviewed in their homes by intensively supervised interviewers. Three years later, in 1995/1996, all respondents were approached for the T2 interview cycle. The participation rates and numbers of decedent respondents are shown in Figure 1. Between T2 and T3 (1998/1999), 342 respondents died. The database of LASA contains contact information about two persons close to the sample member, such as the partner, a child, sibling, or other person who had had close contact. Wherever possible, one proxy respondent was selected, who had been involved in the last three months of the sample member, and who was willing and able to participate. The proxy respondent was approached with a letter with information on the study, followed by a telephone call, to make an appointment for the interview, which was held in the home of the proxy respondent. This research method is known in the literature as “retrospective/after-death approach” or “proxy interview” [20]. The number of proxy respondents amounted to 270, mainly children (50%) and spouses (33%) of the sample members.

### 2.2. Measures

#### 2.2.1. Mood in the Last Week of Life

The interview with the proxy respondent included a one-item question on whether the sample member showed feelings of depression in the last week of life. Scores were 0 (absence of depressed mood) or 1 (presence of depressed mood). Furthermore, the proxy respondents were asked to estimate whether the sample member experienced a sense of peace with the approaching end of life. This was scored as 0 (sense of peace-absent) or 1 (sense of peace present).

#### 2.2.2. Religiousness

Data on *religious affiliation* and *church attendance* were obtained during the first assessment cycle of LASA. Religious affiliation included: Protestant, Roman Catholic, and nonreligious affiliation. The Protestant group consisted of several denominations, but most with origins in the Reformed/Calvinist protestant tradition. The religious affiliation of the parents was also asked, and was coded as: (1) both parents and sample member affiliated; (2) parents affiliated, sample member not affiliated (first generation secularized); (3) neither parents nor sample member affiliated (second generation secularized). Church-attendance was assessed using five response categories, ranging from “once a year or less” (1) to “once a week or more” (5).

The second LASA assessment cycle contained questionnaires on orthodox religious beliefs and salience of religion. The level of adherence to traditional (Christian) religious beliefs was assessed at T2 by the Orthodoxy Scale, which has been regularly used in studies by the Dutch Social and Cultural Planning Office (SCP) [2]. Doctrines included are (asked as “Do you believe in”): life after death, heaven, purgatory, hell, the devil, the actual existence of Adam and Eve, and the Bible as God's word. Answer could be “yes” (score = 1) or “no” (score = 0), yielding a score range of 0–7 (Cronbach's alpha 0.86).

*Salience of religion* was assessed using two items of a religious salience scale [21]: “My religious faith/philosophy of life has a pronounced impact on my daily life” and “When I take important decisions, my religious faith/philosophy of life plays a considerable role.” Response categories range between “totally disagree” (0) to “totally agree” (5). Salience of religion was also probed in the proxy interview, using the same items (“Salience-according-to-proxy”).

#### 2.2.3. Previous Depressive Symptoms Assessed in the LASA Respondent Interview

Depressive symptoms were measured using the Center for Epidemiologic Studies Depression Scale [22]. Subjects were asked how often they experienced each of 20 symptoms during the previous week. The response categories ranged from 0 (“rarely or none of the time”) to 3 (“most of or all the time”), yielding a score range of 0 to 60 (Cronbach *α* = 0.83). A CES-D score of 16 or higher has generally been used as indicative for clinically relevant depressive symptoms, including both minor or subthreshold depression and major depression [23, 24]. Therefore, in the stratified analyses, this cutoff was applied.

#### 2.2.4. Covariates from the LASA Respondent Interview

*Demographic characteristics *of the sample member included age of death, sex, education in years, and marital status (married versus widowed, divorced, or never married).

The number of *major chronic diseases* was assessed at T2, by explicitly asking the respondents whether or not they had or had had any of the following seven conditions: chronic lung disease, cardiac disease, peripheral artery disease, stroke, diabetes mellitus, arthritis, and cancer [25].

#### 2.2.5. Covariates from the Proxy Interview

**Physical State**

The proxy respondents were asked about the presence of serious physical symptoms in the last week of life of the sample member: fatigue, pain, shortness of breath, confusion, and nausea and/or vomiting. Responses (0 “no” and 1 “yes”) were summed to obtain a symptom burden score (range 0–5).

*Cognitive change* between the measurement in 1995/1996 and 3 months before death was assessed using the six-item short form Informant Questionnaire on Cognitive Decline in the Elderly [26]. For every item, the proxy respondent answered on a five-point scale (range 1–5; Cronbach *α* = 0.93). Higher sum scores indicate cognitive decline.

**Time Intervals**

The duration of the periods between the T2 interview and death, and between death and the proxy interview were included to adjust for any influence of time on the outcomes.

Whether the sample members had *expected death* and had been *aware of the approaching end* was estimated by the proxy respondents, with “yes,” “no,” or “more or less” as response categories. When both questions were answered with “yes,” it is assumed that the sample member clearly realized the approaching end.

### 2.3. Statistical Procedure

In the previous paper, associations with the two outcome variables on mood in the last week of life—feeling depressed and sense of peace—were analysed for each of the religious variables, using logistic regression analysis, computing odds ratios (OR) and 95% confidence intervals (95% CI) [1]. Adjustment was made for the covariates with significant associations with the dependent variables, as was evident from prior bivariate and multivariate analyses (also carried out in the stratified subgroups of interest with and without previous depressive symptoms). As there was variation in item nonresponse between the variables, the maximal number of sample members was included in each of the analyses.

Modification of the association with the outcome variables was examined by including the product term between previous depressive symptoms as assessed on T2 (79%) or on T1 (21%, with missing scores at T2) and each of the religion variables in the subsequent logistic regression models. To avoid multicollinearity between first-order terms and product terms, product terms were formed by multiplying the centered (deviation from the mean) scores of both components [27]. The level of statistical significance was set at *P* < 0.05 for main effects, and at *P* < 0.10 for interaction effects, as the power of statistical tests for higher order terms is generally lower than for first order terms [27, 28]. To facilitate interpretation of the interactions, the associations between the religion variables and the outcome variables were examined using logistic regression analyses, adjusted for relevant covariates and stratified for two contrasting subgroups: those who had low levels of depressive symptoms at a previous assessment (CES-D score <16) and those who had high levels of depressive symptoms (CES-D ≥16).

## 3. Results

### 3.1. Characteristics of the Sample

The majority of the sample (Table 1) was male, which is in accordance with the higher expected mortality among males. Mean age of death amounted to 80 years. About one-third was Protestant, one-third Roman Catholic, and one-third nonaffiliated. One-third of the sample members used to attend church on a weekly basis. As examples of items of the orthodoxy, 57% of the sample members reported to believe in heaven, and 30% reported to believe in hell. Salience of religion received higher scores by the sample members at T2, compared to the report by the proxy respondents. The Cohen *κ* for inter-rater agreement was fair for both salience items (.27 and .25). Depressed mood in the last week of life was reported for 28% of the sample members and sense of peace for 76%. 

*Bivariate associations *between covariates and mood in the past week of life have been reported in the previous publication [1]. Depressive symptoms, assessed in previous LASA interviews, significantly predicted the presence of depressed mood in the last week of life (*t* = −2.9, *P* = .005), as well as the absence of a sense of peace (*t* = 4.3, *P* = .000). Neither the duration of the period between the T2 interview and death, nor the duration between death and the proxy interview had significant associations with depressed mood (*t* = 0.6, *P* = .573; *t* = −1.1, *P* = .263) or with sense of peace (*t* = −0.7, *P* = .459; *t* = 0.2, *P* = .869). These time periods did not significantly interact with depressive symptoms assessed in the previous LASA interview and depressed mood or sense of peace in the last week of life (results on request). Similarly, the type of relationship between the respondent and the proxy (whether or not this was the partner or child) did not interact with the association between depressive symptoms and mood in the last week of life (results on request).

Serious physical symptoms and cognitive decline were significantly associated with depressed mood in the last week of life (*t* = −4.2, *P* < .001, and *t* = −2.6, *P* = .009, resp.). In contrast, cognitive decline and higher age were positively associated with a sense of peace (*t* = 2.0, *P* = .043; *t* = 2.3, *P* = .024).

### 3.2. Interactions with Previous Depressive Symptoms

The results of the tests for interactions are shown in Table 2. For depressed mood in the last week of life, previous depressive symptoms significantly interacted with religious affiliation, church-attendance, and orthodox beliefs. For sense of peace, previous depressive symptoms significantly interacted with religious affiliation, church-attendance, and salience according to proxy. The nature of the interactions is illustrated using stratified analyses.  Table 3 summarizes the associations between the religious variables and the proxy's reports on depressed mood and sense of peace in the last week of life, both for those who had high and low CES-D scores at an earlier assessment. Two main patterns emerge. 

First, among those with previous depressive symptoms (CES-D ≥16), there was a significantly higher risk of depressed mood in the last week of life for those who were affiliated with a church, for those who attended church on a more frequent basis, and for those for whom religion was salient according to the proxy respondent. Although only at the level of a statistical trend, the same was found for those with higher orthodoxy scores. No significant association was found between salience of religion and depressed mood in the last week of life.

The second pattern pertains to the other outcome, sense of peace in the last week of life. Here, for those *without* previous depressive symptoms (CES-D <16), there was a higher chance on a sense of peace for those who used to go to church on a regular basis, and for those for whom religion was salient according to the proxy respondent.

### 3.3. Denominational Background

Additional analyses (Table 4) revealed that among those with previous depressive symptoms, the risk of depressed mood was, at trend level, somewhat more evident for Roman Catholics, compared to the nonaffiliated. Among those without previous depressive symptoms, affiliation showed a gradual increase in the likelihood of experiencing a sense of peace in the last week of life; compared to second generation secularized, the difference for the first generation secularized was not significant (OR 2.46), reached trend-level for Roman Catholics (OR 3.15), and was significant for Protestants (OR 3.52). The confidence intervals showed, however, a considerable overlap, indicating that the differences between the three denominational groups do not differ significantly.

## 4. Discussion

The current contribution focused on the role of religiousness in the association between previous depressive symptoms and mood in the last week of life. Information was partly obtained from interviews, as the sample members participated in a prospective population-based study, and partly from after-death interviews with relatives of the deceased sample members.

The previous report on these data showed that there were no significant associations between aspects of religiousness and depressed mood in the last week of life in the full sample [1]. However, the current study shows that among those with *previous* depressive symptoms in the last interview cycle (on average about two years before death), several aspects of religiousness were associated with an increased likelihood of depressed mood in the last week of life: church-attendance, (Roman Catholic) church-membership, and salience of religion (salience-according-to-proxy). 

In contrast, among those *without* previous depressive symptoms, church-attendance, church-membership, and salience of religion (salience-according-to-proxy) were associated with a higher likelihood of a sense of peace with the approaching end of life. This sense of peace had the lowest reports among the nonchurch members with nonaffiliated parents (second generation secularized).

The finding that religiousness is associated with depressed mood in the last week of life for those who had previous depressive symptoms, at least at the level of subthreshold depression, is appealing and raises the question what mechanism is at work. In discussing theories on death anxiety, Kastenbaum considers that death concern belongs to the core Christian conceptions [29]. He states that it is possible that Christian doctrine may intensify both anxiety, living in dread of judgment, and serenity, even with longing and impatience. Kastenbaum calls for more elaborate empirical research to understand the psychological and social key factors through which individuals and families come to terms with both the “dread” and the “welcome” of Christian death. The current results seem to offer empirical support for the existence of both positions. 

Church-attendance showed the greatest contrast between those with and without previous depressive episodes. The level of orthodoxy of Christian beliefs showed a contrast as well, but was significant only at the level of a statistical trend in those with previous depressive symptoms. Thus, the cognitive (doctrinal) aspects of religiousness do not seem to represent the main explanation, whereas behavioural and motivational aspects (as measured with church-attendance and salience of religion) do come to the fore. The emotional facet, the feeling of being accepted by the deity, or abandoned, could be even more central. Especially feelings of abandonment by God are known to have high correlations with depression, and, hypothetically, may result in a deeper crisis when the belief in sustainment by the deity seems to be out of grasp [30]. Future research should also address the emotional facet of religiousness in this context.

In the current sample of older adults, there was still considerable membership of religious denominations, but the nonaffiliated represented a sizable group. About one-third of this group had nonaffiliated parents as well. The main impression is that the nonaffiliated had the least disadvantages (least depressed mood in the last week of life) in case of previous depressive symptoms. On the other hand, for those without previous depressive symptoms, the nonaffiliated who had nonaffiliated parents as well had the least advantages with respect to sense of peace. Perhaps, this group may no longer have access to the supportive aspects of religious faith, in contrast to the first generation secularised. It should however be noted that the denominational differences in the current study were statistically modest or even weak, because of small group numbers in the stratified analyses.

The contemporary society of The Netherlands should be characterized as highly secularized, and in younger samples, the first-generation secularized represents a large group. Some convictions and remnants of doctrines may persist, along with spiritual feelings, rituals, and new beliefs, such as feelings about reincarnation. With respect to the needs and strengths of secularized older adults when facing death, future research may include aspects of spirituality and other dimensions of meaning in life. For those who have been raised within a religious tradition, inclusion of measures of intrinsic and extrinsic religious motivation is warranted, as well as deeper inquiry into the contents of beliefs, to reveal which church doctrines and which motivations are more sustaining and supportive and which are more depressing.

Salience of religion as reported by the proxy respondent, but not salience as reported by the sample member, was associated with a sense of peace in the last week of life. One may therefore assume that the proxy respondents (especially the spouses) used to have similar religious beliefs and practices as the respondents had during their lives. On the one hand, the results from the current study might show how religiousness of the proxy respondent helped to cope with the loss of the relative. On the other had, the considerable degree of personal involvement of the proxy respondents may have influenced the results. The current study focuses, however, on effect modification by a variable that was assessed earlier in life: previous depressive symptoms. Apparently, the global assessment of mood in the last week of life by the proxy respondents did not prevent that more or less opposing results for the nondepressed and depressed still could be described.

One limitation of the current study is that mood in the last week of life was not directly observed in the respondents when they were terminally ill, but was assessed retrospectively. For the current sample, Klinkenberg and colleagues verified some information obtained from the proxy respondents with reports from physicians [31]. The proxy respondents seemed to provide accurate information with respect to chronic physical conditions. As Addington-Hall and McPherson (2001) point out in their review about the validity of after-death interviews, some studies provided evidence that there is little correspondence between the sample member and the proxy respondent about topics like depressed mood [32]. According to research on the concordance of patient and caregiver reports, both patient and caregiver depression were common predictors of disagreement [33]. Although the results of the current study closely examined for effects of previous depressive symptoms, further reasons for nonconcordance could not be ruled out. The same is true for recall bias by the proxy respondents, many of whom were interviewed after two years. An important concern regarding research about the end of life in the general population remains the difficulty of timely identification of respondents, and if they can be identified, few will be able or allowed to participate. Interviews with surviving relatives therefore will remain a source of knowledge about the last phase of life. Another limitation is that both outcomes consisted of one-item measures. One recommendation for future research is to examine the state of mood more fully. Meanwhile, psychometrically acceptable measures of the quality of the dying experience have become available, such as two versions of the quality of dying in long-term care instrument [34]. A related point is that the current study did not include measures on, partly overlapping, concepts such as spiritual distress, or death anxiety [6, 35]. Measures on emotional aspects of religiousness, spirituality, and secular sources of meaning in life may be included in further research.

The current results suggest that vulnerability to depression is an important aspect for the direction of the relationship between religiousness and mood in the last phase of life. It should be underlined that replication is desirable, both employing quantitative and qualitative research methods, before any recommendations can be done to professionals in the field of palliative care. Recent guidelines and recommendations for the quality of spiritual care—as a dimension of palliative care—provide suggestions for screening questions to assess spiritual life in patients in palliative care, to keep an eye on spiritual distress or religious struggle, and how to integrate spiritual issues into the treatment plan [36]. Verifying the recent history of depressive symptoms may provide a cue to detect any religious struggle or other severe existential doubts, which may possibly represent an additional burden for those in the last phase of life.

## Figures and Tables

**Table 1:** Characteristics of deceased sample members of the Longitudinal Aging Study Amsterdam (LASA) between 1995 and 1998.

	*n*	Range	Mean	(SD)	%
Sex (% female)	270				38.1
Age of death	270	59–91	80.4	(7.5)	
Time interval: last respondent interview–death (days)	269	8–1321	589	(330)	
Time interval: death proxy interview (days)	270	131–1479	789	(316)	

*Last* * respondent interview*					

Marital state (% married)	263				47.1
Education (years)	263	5–18	8.6	(3.4)	
Number of major chronic diseases	270	0–7	1.6	(1.2)	
Depressive symptoms (% ≥16) [*n* = 56 at T1, *n* = 211 at T2]	267	0–44	10.4	(8.6)	21.7
Religious affiliation	270				
Protestant					31.9
Roman Catholic					28.9
Other					1.5
Nonaffiliated^(2)^					37.8
Church attendance in 1992 (LASA baseline interview)	270	1–5	2.7	(1.8)	
Orthodoxy scale	203	0–6	2.9	(2.3)	
Salience of religion, last interview with sample member	203	0–8	5.1	(2.1)	

*Interview with proxy respondent*					

Cognitive decline *according to proxy respondent * ^(1)^	238	1–5	3.8	(0.8)	
Serious physical symptoms in the last week of life^(1)^	259	0–5	2.2	(1.3)	
Mood in last week of life *according to proxy respondent *					
Depressed mood	233				28.2
Sense of peace—absent	204				23.5
Salience of religion *according to proxy respondent *	268	0–8	3.5	(3.1)	
Expected death/aware of approaching end (both “yes”, %)	270				53.0

^(1)^High scores indicate more problems.

^(2)^Among the nonaffiliated: one or both parent(s) affiliated 61% (*n* = 59) and both parents nonaffiliated 39% (*n* = 38) (9 had missing value).

**Table 2:** Interaction effects between previous depressive symptoms and aspects of religiousness for mood (depressed mood or sense of peace) in the last week of life, according to proxy respondents; results of logistic regression analyses; results printed in bold are statistically significant.

	Depressed mood in last week of life (according to proxy)				Sense of peace with approaching end of life (according to proxy)			
	interaction with previous depressive symptoms (CES-D)^(a)^				interaction with previous depressive symptoms (CES-D)^(b)^			
	*N*	B (SE)	Wald	*P*	*N*	B (SE)	Wald	*P*
Church members versus nonaffiliated	216	**0.08** **(0.04)**	**3.8**	**.050**	192	−0.13** (0.05)**	**7.9**	**.005**
Church attendance	218	**0.02 (0.01)**	**3.6**	**.058**	194	−0.03**(0.01)**	**6.8**	**.009**
Orthodox beliefs	162	**0.03 (0.01)**	**5.6**	**.018**	156	−0.01 (0.01)	0.2	.644
Salience of religion	163	0.01 (0.01)	0.3	.615	154	−0.01 (0.02)	0.5	.482
Salience according to proxy	217	−0.01 (0.01)	0.2	.650	193	−0.01** (0.01)**	**3.4**	**.067**

^(a)^Adjusted for effects by physical distress (according to proxy respondent) and cognitive decline (according to proxy respondent).

^(b)^Adjusted for effects by age of death, physical distress (according to proxy respondent), and expectance of death/awareness of the approaching end (according to proxy respondent).

**Table 3:** Mood in the last week of life, as reported by proxy respondents of deceased sample members of the Longitudinal Aging Study Amsterdam between 1995 and 1998: associations with aspects of religiousness, stratified for previous depressive symptoms.

		Depressed mood in last week of life (according to proxy)^(a)^					Sense of peace with approaching end of life (according to proxy)^(b)^				
		*N*	Wald	*P*	OR	95% CI	*N*	Wald	*P*	OR	95% CI
Previously nondepressed	Church members versus nonaffiliated	171	0.1	.739	0.88	0.42–1.86	151	2.3	.129	1.92	0.83–4.45
	Church attendance [1–5]	173	0.0	.942	1.01	0.83–1.23	153	**4.5**	**.034**	**1.31**	**1.02**–**1.68**
	Orthodox beliefs [0–6]	131	0.1	.770	0.97	0.81–1.17	123	0.6	.433	1.08	0.89–1.33
	Salience of religion [0–8]	131	0.8	.360	0.92	0.76–1.11	124	1.3	.250	1.14	0.91–1.41
	Salience according to proxy [0–8]	173	0.1	.707	1.02	0.91–1.15	152	**4.1**	**.042**	**1.15**	**1.00**–**1.32**

Previously depressed (CES-D ≥ 16)	Church members versus nonaffiliated	45	**4.9**	**.028**	**9.05**	**1.28**–**64.02**	41	0.7	.412	0.51	0.10–2.56
	Church attendance [1–5]	45	**6.4**	**.011**	**2.32**	**1.21**–**4.44**	41	1.1	.300	0.79	0.51–1.23
	Orthodox beliefs [0–6]	32	*3.5*	*.060*	*1.58*	*0.98*–*2.55 *	30	1.1	.299	1.24	0.83–1.86
	Salience of religion [0–8]	32	1.6	.207	1.41	0.83–2.40	30	1.8	.174	0.68	0.39–1.18
	Salience according to proxy [0–8]	44	**4.0**	**.046**	**1.40**	**1.00**–**1.93**	41	0.4	.540	0.92	0.70–1.20

Results printed in **bold** are statistically significant (*italics*: trend).

^(a)^Adjusted for effects by physical distress (according to proxy respondent) and cognitive decline (according to proxy respondent).

^(b)^Adjusted for effects by age of death, physical distress (according to proxy respondent), and expectance of death/awareness of the approaching end (according to proxy).

**Table 4:** Religious affiliation and mood in the last week of life, as reported by proxy respondents of deceased sample members of the Longitudinal Aging Study Amsterdam between 1995 and 1998: stratified for previous depressive symptoms.

		Depressed mood in last week of life (according to proxy)^(b)^					Sense of peace with approaching end of life (according to proxy)^(b)^				
		*N*	Wald	*P*	OR	95% CI	*N*	Wald	*P*	OR	95% CI
Previously nondepressed	Protestant^(a)^	57	1.4	.245	0.52	0.17–1.57	47	**4.3**	**.039**	**3.52**	**1.07**–**11.58**
	Roman Catholic^(a)^	58	0.0	.885	0.93	0.32–2.65	49	*3.8*	*.053*	*3.15*	*0.99*–*10.08 *
	First-generation secularized^(a)^	29	0.1	.774	1.17	0.40–3.48	35	2.1	.144	2.46	0.74–8.25
	Second-generation secularized	17			1		21			1	

Previously depressed (CES-D ≥ 16)	Protestant^(a)^	15	0.5	.502	1.78	0.33–9.55	14	0.1	.718	1.47	0.18–11.72
	Roman Catholic^(a)^	11	*2.8*	*.095*	*4.67*	*0.77*–*28.47 *	12	1.5	.220	0.27	0.04–2.11
	First-generation secularized^(a)^	11	0.0	.999	1.00	0.15–6.53	9	0.4	.518	0.50	0.06–4.09
	Second-generation secularized	11			1		7			1	

Results printed in **bold** are statistically significant (*italics*: trend).

^(a)^Reference group is second generation secularized (nonaffiliated respondents with nonaffiliated parents).

^(b)^Nonadjusted because of too low number of respondents; when adjusted as in Table 3, the results are slightly stronger but with problematic wide 95% CI's.
